# Six years’ experience with LipidSeq: clinical and research learnings from a hybrid, targeted sequencing panel for dyslipidemias

**DOI:** 10.1186/s12920-020-0669-2

**Published:** 2020-02-10

**Authors:** Jacqueline S. Dron, Jian Wang, Adam D. McIntyre, Michael A. Iacocca, John F. Robinson, Matthew R. Ban, Henian Cao, Robert A. Hegele

**Affiliations:** 1 Robarts Research Institute, Schulich School of Medicine and Dentistry, Western University, 1151 Richmond St, London, ON N6A 5B7 Canada; 2 Department of Biochemistry, Schulich School of Medicine and Dentistry, Western University, 1151 Richmond Street, London, ON N6A 5B7 Canada; 3 Department of Biomedical Data Science, Stanford School of Medicine, Stanford University, 450 Serra Mall, Stanford, CA 94305 USA; 4 Department of Medicine, Schulich School of Medicine and Dentistry, Western University, 1151 Richmond St, London, ON N6A 5B7 Canada

**Keywords:** Targeted next-generation sequencing panel, Familial hypercholesterolemia, Hypertriglyceridemia, Dyslipidemia, Metabolic disorder, Lipid, Lipoprotein

## Abstract

**Background:**

In 2013, our laboratory designed a targeted sequencing panel, “LipidSeq”, to study the genetic determinants of dyslipidemia and metabolic disorders. Over the last 6 years, we have analyzed 3262 patient samples obtained from our own Lipid Genetics Clinic and international colleagues. Here, we highlight our findings and discuss research benefits and clinical implications of our panel.

**Methods:**

LipidSeq targets 69 genes and 185 single-nucleotide polymorphisms (SNPs) either causally related or associated with dyslipidemia and metabolic disorders. This design allows us to simultaneously evaluate monogenic—caused by rare single-nucleotide variants (SNVs) or copy-number variants (CNVs)—and polygenic forms of dyslipidemia. Polygenic determinants were assessed using three polygenic scores, one each for low-density lipoprotein cholesterol, triglyceride, and high-density lipoprotein cholesterol.

**Results:**

Among 3262 patient samples evaluated, the majority had hypertriglyceridemia (40.1%) and familial hypercholesterolemia (28.3%). Across all samples, we identified 24,931 unique SNVs, including 2205 rare variants predicted disruptive to protein function, and 77 unique CNVs. Considering our own 1466 clinic patients, LipidSeq results have helped in diagnosis and improving treatment options.

**Conclusions:**

Our LipidSeq design based on ontology of lipid disorders has enabled robust detection of variants underlying monogenic and polygenic dyslipidemias. In more than 50 publications related to LipidSeq, we have described novel variants, the polygenic nature of many dyslipidemias—some previously thought to be primarily monogenic—and have uncovered novel mechanisms of disease. We further demonstrate several tangible clinical benefits of its use.

## Background

Dyslipidemias, defined as extreme deviations of plasma lipids or lipoproteins, are commonly encountered clinically [1]. They are often associated with increased risk of cardiovascular disease and other complications such as acute pancreatitis [2, 3]. There are 25 monogenic dyslipidemias caused by variants in 25 genes [1, 4, 5], most of which were identified > 10 years ago using classical biochemical and genetic mapping methods [6]. With the exception of heterozygous familial hypercholesterolemia (FH), monogenic dyslipidemias are rare disorders [1] and can sometimes display multisystem syndromic features [1, 4]. Most show recessive inheritance and typically result from pathogenic rare variants—either single-nucleotide variants (SNVs) or copy-number variants (CNVs)—in well-established causal genes. Further, some dyslipidemias are polygenic, resulting from contributions of several types of genetic determinants including incompletely penetrant rare variants and small-effect common variants [2, 7]. Accumulated variants within an individual’s genome can predispose to more severe phenotypic expression [7]. In addition to genetic determinants, several secondary factors—diet, obesity, activity level, other medical conditions such as diabetes or hypothyroidism, and certain medications—can exacerbate the clinical presentation of both monogenic and polygenic dyslipidemias [8, 9].

For over 25 years, our laboratory has studied both monogenic and polygenic dyslipidemias. Patient care and genetic analysis have coexisted through fortuitous geographic convergence of our lipid clinic, genetics research laboratory and genomic core facility, and through uninterrupted funding for a genetics research program. At the time the clinic and research program were established, our ethics review panel stipulated that genetic results were to be shared with patients, and this became our practice. Patient samples come from both local clinical practices and international colleagues; virtually all patients seen in the clinic have consented to provide DNA samples for research. Between 1998 and 2012, DNA analysis was performed by automated Sanger sequencing. In 2013, we transitioned to next-generation sequencing using the custom-designed “LipidSeq” panel; results from the latter are reported here.

Because our clinical practice spans all dyslipidemias, we have focused on their ontology [1, 10, 11] and on documenting dyslipidemia-associated gene variants [11]. Our molecular classification system ultimately informed the design of the LipidSeq panel for genes underlying monogenic dyslipidemias [12, 13]. We also designed the panel to target genes causing monogenic disorders for which dyslipidemia is a secondary manifestation, such as inherited forms of diabetes. A benefit of the high depth of coverage afforded by our panel is the ability to concurrently identify CNVs along with SNVs. Furthermore, our longstanding interest in the polygenic basis of plasma lipids [14–16] motivated us to simultaneously genotype common single-nucleotide polymorphisms (SNPs) [17]. We easily accommodated 185 “micro-sequencing” reactions to genotype lipid trait-associated SNPs from the Global Lipid Genetics Consortium genome-wide association studies (GWAS) of plasma lipids [17–19].

Thus, LipidSeq is a hybrid panel that detects: 1) functionally relevant rare SNVs and CNVs in genes underlying monogenic dyslipidemias, and 2) common variants, particularly SNPs, that we use to build polygenic scores [20]. Since 2014, LipidSeq results have helped clarify the genetic basis for hundreds of dyslipidemic patients and have been reported in > 50 peer-reviewed publications. In this report, we briefly describe our aggregated research findings and discuss the clinical benefit of our LipidSeq panel.

## Methods

### The LipidSeq panel

LipidSeq was designed for clinical resequencing of genomic loci associated with dyslipidemia and related metabolic traits [4, 12]. It targets exons plus 250 bp into each flanking intron and the 5′- and 3′-untranslated regions of 69 genes, including: 1) 25 causative genes for monogenic dyslipidemias; 2) 16 causative genes for inherited lipodystrophies; 3) 13 for subtypes of maturity-onset diabetes of the young (MODY) and inherited diabetes; and 4) 15 candidate genes in lipoprotein metabolism for which no pathogenic mutations have yet been found (Additional file 1: Table S1). LipidSeq also “micro-sequences” the area surrounding 185 GWAS SNPs, from which we use certain SNP subsets to build small-scale polygenic scores for low-density lipoprotein (LDL) cholesterol, triglyceride, and high-density lipoprotein (HDL) cholesterol [20]. An expanded rationale for the LipidSeq design are reported elsewhere [4], as well as quality assessment and validation of the panel [12].

### Clinic samples

**Fig. 1:**
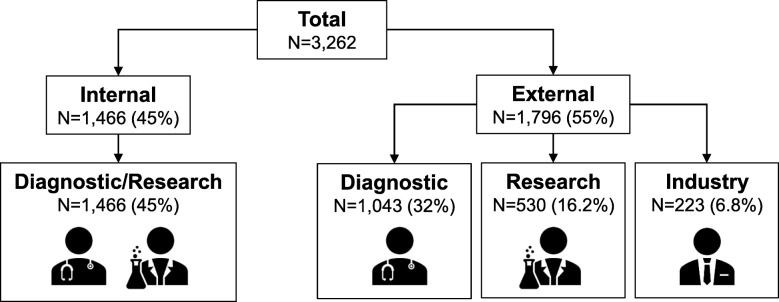
Origin of samples sequenced with the LipidSeq panel. Internal samples (45%) come from patients who were referred to the Lipid Genetics Clinic for clinical care and provided consent to have their DNA sequenced. External samples (55%) are referred from all over the world for various reasons. 32% of samples are externally referred from clinical colleagues and are single patient or nuclear family samples sent for diagnosis, typically because they lack access or ability to pay for commercial testing. Each external patient or substitute decision-maker reviews the approved letter of information with the genetics clinic coordinator by telephone or Skype before providing consent. Another 16.2% of samples are sent for external research purposes, typically through academic collaborations; protocols and consent follow in accordance with the collaborating institution. The remaining 6.8% of samples are referred from industry, usually contracted by pharmaceutical companies requesting baseline molecular characterization of participants in clinical trials of investigational lipid-lowering therapies

**Fig. 2:**
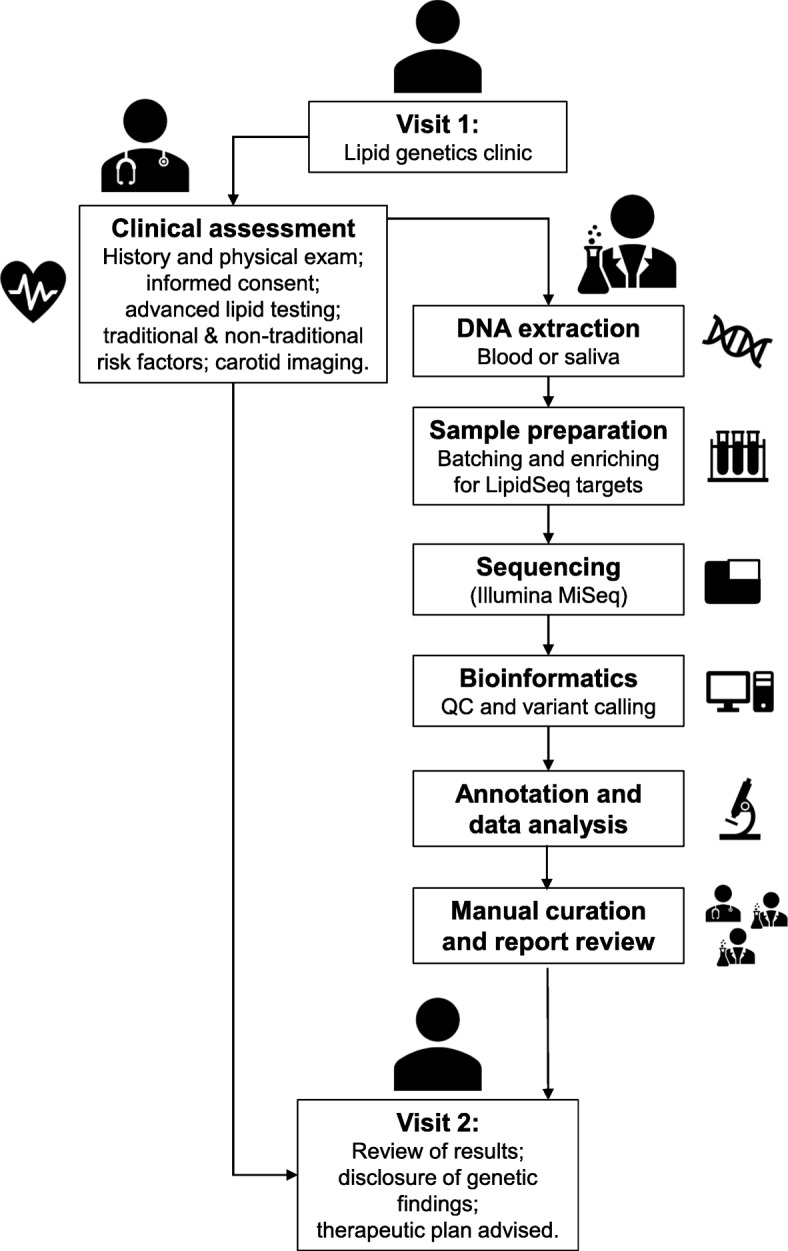
Overview of the patient and DNA sample journeys. Upon arrival to clinic (Visit 1), the patient undergoes a clinical assessment (left branch). During their clinic visit, blood is drawn for subsequent lipid tests, as well as genetic assessment (right branch). After DNA has been extracted and has undergone sequencing and bioinformatic processing, genetic factors that are relevant to the patient’s phenotype or present as risk factors for future health concerns are relayed back to the patient at a follow-up appointment. During the follow-up appointment (Visit 2), an additional clinical assessment is performed if required. Advice is given by combined clinical parameter with genetic results, if appropriate

Figure 1 shows the breakdown of samples studied using LipidSeq. The Lipid Genetics Clinic at the London Health Sciences Centre, University Hospital (London ON, Canada) was established in 1998 and operates a half-day each week, serving a region with a population of ~ 2 million people and providing care to outpatients referred from family practitioners and specialists. Because the main clinic physician (R.A.H.) also holds peer-reviewed research funding to study genetics of dyslipidemia and related disorders, patients are invited to provide DNA samples for research. The journey of a patient and their consented DNA sample are outlined in Fig. 2. On initial consultation (Visit 1), after taking a medical history and performing a physical examination, informed consent is obtained and the patient provides a fasting blood sample for: 1) determination of routine lipid profile (total, LDL and HDL cholesterol, and triglyceride); 2) advanced lipid profiling (including apolipoprotein [apo] B and A1, and lipoprotein [a]); 3) screening for secondary causes of dyslipidemia (including hypothyroidism, and hepatic and renal conditions); 4) screening non-traditional cardiovascular risk markers (including high sensitivity C-reactive protein and carotid intima-medial thickness); and 5) DNA extraction. After sample processing and reporting, results are discussed with the patient at Visit 2. The project was first approved in 1998 by the Western University ethics review board (#07290E) and has been updated and renewed continuously.

### External samples

We also accept patient samples referred from colleagues provided consent is obtained following appropriate institutional standards. We also receive contracted samples from pharmaceutical companies to genotype de novo or validate previous diagnostic results for clinical trials. External samples follow the same processing flow as internal samples (Fig. 2).

### DNA extraction and isolation

Genomic DNA is extracted from blood (95% of samples) using the Puregene® DNA Blood Kit (Gentra Systems, Qiagen Inc., Mississauga ON, Canada) (*Cat No. 158389*) or saliva (5% of samples) using the Oragene DNA kit (DNA Genotek Inc., Ottawa ON, Canada; *Cat No. OG-500*).

### Sample preparation and sequencing

DNA samples prepared in batches of 24 are indexed and enriched using the Nextera® Rapid Capture Custom Enrichment Kit (*Cat No. FC-140-1009*) “LipidSeq” design [12]. Sequencing is performed for each batch at the London Regional Genomics Centre (www.lrgc.on.ca; London ON, Canada) on an Illumina MiSeq personal sequencer (Illumina, San Diego CA, USA).

### Bioinformatic processing and quality assurance

Paired FASTQ files are generated for each sample after sequencing and are imported into CLC Bio Genomics Workbench (CLC Bio, Aarhus, Denmark) for bioinformatic processing. Sequencing reads are aligned to the human reference genome (hg19/GRCh37) and undergo local realignment to improve alignment quality. From the assembled reads, variants are called if there are discrepancies between the reference genome and the sample’s sequence. Following this, VCF and BAM files are created for each sample; these files contain information on the genomic position and zygosity of identified variants, as well as the depth of coverage for each sequencing read. A detailed explanation of the bioinformatic and quality assurance processes have already been reported [21].

### Data analysis

The initial tool used for the annotation and analysis of variants was the open-sourced tool, ANNOVAR [22]. Recently, we have switched to the commercially available software, VarSeq® (Golden Helix, Inc., Bozeman MT, USA) for variant annotation and analysis. Our software upgrade allowed us to assess for CNVs, which was not previously accessible using ANNOVAR alone.

#### Single-nucleotide variants and indels

Rare variants with potential for protein-altering effects are of primary interest. We consider variants with a minor allele frequency of ≤1% or absent from publicly available genotype databases. Our reference database has changed over the years as more comprehensive databases became available; starting with the 1000 Genomes Project (http://browser.1000genomes.org/index.html) [23], we progressed to the Exome Aggregation Consortium (ExAC; http://exac.broadinstitute.org/) [24], and finally to the Genome Aggregation Database (gnomAD; https://gnomad.broadinstitute.org/) [25]. Only rare variants that impact the amino acid sequence or canonical splice sites are considered, including missense, nonsense, insertions or deletions (indels), splice-donor, and splice-acceptor variants; the rationale for this is that a change to the encoded protein will likely have a phenotypic impact. In an attempt to avoid benign variants, multiple in silico prediction tools are used to identify rare variants with possible damaging or deleterious effects, including Combined Annotation Dependent Depletion (CADD; http://cadd.gs.washington.edu/score) [26, 27], Polymorphism Phenotyping version 2 (PolyPhen2; http://genetics.bwh.harvard.edu/pph2/) [28], Sorting Intolerant From Tolerant (SIFT; http://sift.jcvi.org/) [29], and MutationTaster (http://www.mutationtaster.org/) [30]. Since its introduction in 2015, we also consider ACMG classifications for each variant of interest, and are in the process of reannotating our entire variant database using these criteria [31]. We have been utilizing Franklin by Genoox (https://franklin.genoox.com/home), a web tool for variant interpretation for this process.

#### Copy-number variants

CNVs are detected using the VarSeq-CNV® caller algorithm. Using BAM files, this algorithm detects differences in read depth of a sample compared to a group of “reference” samples without CNVs. More details of this method and our standard parameters were reported previously [32].

#### Polygenic scores

We calculate small polygenic scores using lipid-altering alleles from a subset of SNP loci captured by our panel; 10, 16, and 9 SNPs comprise the LDL cholesterol, triglyceride and HDL cholesterol scores, respectively. All targeted SNPs were reported by the Global Lipids Genetics Consortium as having a statistically significant association with at least one of the three lipid traits [17–19]. Our weighted polygenic score calculation considers the total number of trait-raising alleles at a single locus (0, 1, or 2) multiplied by that allele’s beta coefficient determined from GWAS [17–19]. Each product is summed to produce the overall weighted polygenic score for the trait. A more detailed explanation behind polygenic scores and their calculations is available [7]. Each individual sequenced by LipidSeq has polygenic scores calculated for each of LDL cholesterol, triglyceride and HDL cholesterol, regardless of their referral phenotype. An extreme accumulation of common SNPs was defined as an extreme polygenic score, classified as a score greater than or equal to the 90th percentile previously determined using a normolipidemic reference group [7].

### Reporting clinically relevant genetic determinants of interest

The preliminary list of computationally prioritized rare variants from each patient sample—either SNVs, indels, or CNVs—with potentially damaging or deleterious effects derived from the pipeline is first checked and reviewed manually by two laboratory personnel (A.D.M. and J.W.). Polygenic scores are also shown on the draft patient report. Prior to Visit 2, the patient’s draft report is reviewed by the laboratory scientist (A.D.M.) and the physician (R.A.H.) before finalizing the report of both rare variant results and polygenic scores, with interpretations derived by consensus from the three reviewers (A.D.M., J.W. and R.A.H.). We put particular emphasis on rare variants disrupting genes with a direct relation to the phenotype of interest. With respect to polygenic scores, for brevity and simplicity, we only report to the patient the score associated with their referral phenotype (i.e. a patient with hypertriglyceridemia will only receive results from the triglyceride risk score). Based on a patient’s report, they can receive either: a) a genetically based diagnosis; b) a genetic confirmation of a previously received diagnosis; c) a “relevant” genetic result (i.e. a variant that has not been functionally confirmed to cause the phenotype, but is still predicted to be damaging and occurs within a phenotypically associated gene); or d) a negative result, indicating that we were unable to identify any sort of genetic determinant related to the phenotype. Each report is proofread and signed by the laboratory director (R.A.H.). Hard copies of reports for Lipid Genetics Clinic patients are added to patient paper charts and the findings are reported to the patient at Visit 2. Hard copies of reports for externally referred samples are mailed to the referring physician.

## Results

### Characterization of sequenced samples

**Fig. 3:**
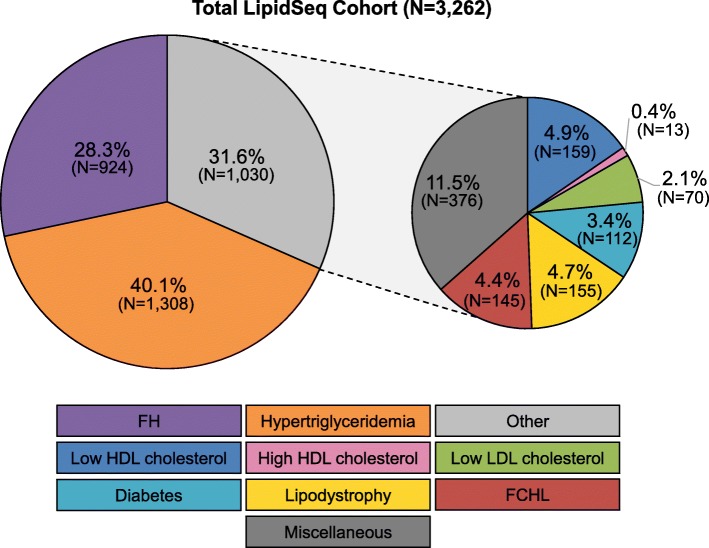
Breakdown of phenotypes from samples sequenced with the LipidSeq panel. The most prevalent phenotypes include FH and hypertriglyceridemia, accounting for ~ 70% of total samples. The remaining ~ 30% of samples are a mix of dyslipidemia and other metabolic phenotypes. Abbreviations: *FH* familial hypercholesterolemia, *FCHL* familial combined hyperlipidemia, *HDL* high-density lipoprotein, *LDL* low-density lipoprotein

To date, we have sequenced 3262 samples from both internal and external sources (Fig. 1), of which 1466 (45.0%) were from the Lipid Genetics Clinic and 1796 (55.0%) were received from external sources for diagnostic (32.0%), research (16.2%), and industry-contracted (6.8%) purposes. Demographic and clinical information from our cohort of internal patient samples is presented in Table 1. The phenotypic breakdown of our total sample cohort is illustrated in Fig. 3.

**Table 1:** Clinical and demographic information on internal subject samples

	Males	Females
N	862	604
Age	47.9 ± 15.0	49.1 ± 16.4
BMI (kg/m^2^)	29.7 ± 5.61	28.6 ± 6.67
Total cholesterol (mmol/L)	6.37 ± 6.33	6.66 ± 2.86
Triglyceride (mmol/L)	5.69 ± 9.39	4.03 ± 7.87
HDL cholesterol (mmol/L)	1.03 ± 0.36	1.38 ± 0.53
LDL cholesterol (mmol/L)	3.30 ± 1.72	3.95 ± 1.81

Values are indicative of the mean ± SD. Data values are missing from each clinical category. Values were taken from the earliest visit. To convert from mmol/L to mg/dL for cholesterol, multiply by 38.67. To convert from mmol/L to mg/dL for triglyceride, multiply by 88.57. Abbreviations: *BMI* body-mass index, *HDL* high-density lipoprotein, *LDL* low-density lipoprotein

The most prevalent phenotype is hypertriglyceridemia (40.1%), followed by FH (28.3%). Briefly, patients with hypertriglyceridemia have elevated triglyceride levels (≥ 1.8 mmol/L) and can present with different clinical features depending on whether the patient has a mild-to-moderate (> 1.8 and < 10 mmol/L) or severe (≥ 10 mmol/L) deviation [33, 34]. These patients are referred to clinic to identify a possible genetic basis for their condition, and for recommendation of treatment options. In contrast, patients referred with “FH” have high prior clinical suspicion of this condition, mainly due to extremely elevated LDL cholesterol levels (> 5.0 mmol/L). With our LipidSeq panel, we are often able to provide a genetic confirmation of the FH diagnosis, which in turn may support the use of more intensive therapeutic strategies to lower LDL cholesterol levels and decrease risk for cardiovascular disease.

The remaining 31.6% of samples include: low or high HDL cholesterol levels (i.e. hypo- and hyperalphalipoproteinemia, respectively), low LDL cholesterol levels (hypobetalipoproteinemia/abetalipoproteinemia), familial combined hyperlipidemia, diabetes, lipodystrophy, and miscellaneous conditions including elevated levels of lipoprotein(a), atypical hyperlipidemia, and severe obesity (Additional file 1: Table S2). Patients referred with low HDL cholesterol levels may be at an increased risk for cardiovascular disease [35–37]. Conversely, patients with high HDL cholesterol levels were previously thought to be at a decreased risk for cardiovascular disease; however, in some instances the causative molecular mechanism increases risk due to impaired clearance of HDL particles [38]. Patients with familial combined hyperlipidemia have elevations of both LDL cholesterol and triglyceride levels, which increases risk for cardiovascular disease. Meanwhile, patients with diabetes, insulin resistance, or uncontrolled glucose are usually referred to clinic for assistance in management of the dyslipidemic component of their phenotype. Patients referred to us with a clinical suspicion of partial lipodystrophy are often able to receive a genetic confirmation of this diagnosis. Meanwhile, patients with lipoprotein(a) levels in the top 5th percentile of the population (i.e. ≥ 36 mg/dL) are referred to our clinic for assistance in managing modifiable cardiovascular risk factors, since no treatment is presently available. “Atypical hyperlipidemia” is used to describe patients with multiple perturbations of lipid variables that do not fit in the “familial combined hyperlipidemia” category. Patients referred due to severe obesity often have dyslipidemia and diabetes-related complications requiring management.

### Rare variant analysis

**Fig. 4:**
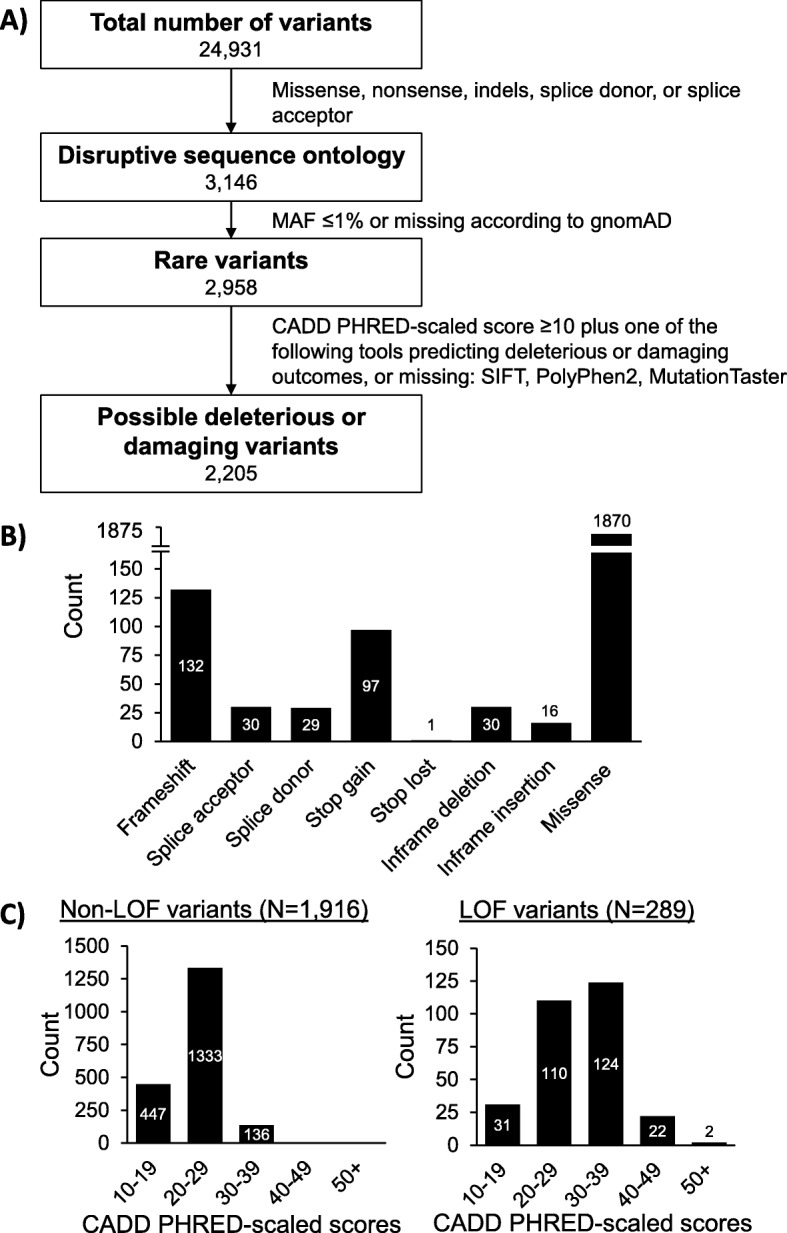
Breakdown of unique rare variants across 3262 samples sequenced. **a** This flowchart demonstrates the number of unique variants that are filtered out at each progressive stage of our rare variant analysis algorithm. A total list of annotated variants is available in Additional file 1: Table S3. **b** The ontology breakdown of 2205 possible deleterious or damaging variants is presented in this bar graph. Loss-of-function variants are considered to be those with ontologies of either frameshift, splice acceptor, splice donor, stop gain, or stop loss. **c** These bar graphs demonstrate the distribution of CADD PHRED-scaled scores for 1916 non-loss-of-function variants (left) and 289 loss-of-function variants (right). Abbreviations: *indels* insertions or deletions, *LOF* loss-of-function, *MAF* minor allele frequency

A total of 24,931 unique variants were identified across 3262 samples sequenced with LipidSeq (Additional file 1: Table S3). After applying our rare variant filtering criteria (Fig. 4a), 2205 variants were of potential interest based on their disruptive sequence ontology and predictions of producing a deleterious or damaging protein product. Of these, 289 are predicted to be loss-of-function variants based on ontologies of either frameshift, splice acceptor, splice donor, nonsense (stop gain), or stop loss (Fig. 4b). After filtering these variants further based on a stricter CADD PHRED score of ≥20, 258 variants would likely be of clinical interest given their predicted level of having a damaging effect (Additional file 1: Table S4), especially if the variant disrupts a gene with a known relationship to the patient’s phenotype (ex. an *LDLR* variant in a patient with FH). Lastly, from our rare variant findings, we identified 191 total CNVs, of which 77 were unique (Table 2). The distributions of CADD PHRED-scaled scores for all variants predicted to be possibly deleterious or damaging are presented in Fig. 4c.

**Table 2:** Unique CNVs observed identified across 3262 samples using the LipidSeq panel

Gene	CNV state	Regions affected	Instances observed	Related publication
*ABCA1*	5’UTR – 3’UTR	Deletion (het)	1	[39]
	Exons 47–48	Deletion (het)	1	
	Exons 8–31	Deletion (het)	2	[39]
	Exons 4–7	Duplication	1	
	Exon 4	Deletion (het)	1	[39]
*ABCG1*	Non-coding exon 1–3’UTR	Duplication	2	
*ABCG8*	Exons 4–6	Duplication	3	
*AGPAT2*	Exons 2–4	Deletion (het)	1	
	5’UTR – exon 1	Deletion (het)	1	
*ANGPTL3*	Exon 3–3’UTR	Deletion (het)	1	
*APOA5*	5’UTR – 3’UTR	Deletion (het)	1	
*APOA5* and *APOA4*	5’UTR – 3’UTR, 5’UTR – 3’UTR	Duplication	1	
*APOB*	5’UTR – 3’UTR	Duplication	1	
*APOC2*	Non-coding exon 1	Deletion (hom)	1	
*BLK*	Exon 2–3’UTR	Duplication	29	
	Exon 10	Duplication	1	
*CAV2*	5’UTR – exon 1	Duplication	1	
*CETP*	5’UTR – exon 2	Deletion (het)	1	
*CIDEC*	Exon 4–3’UTR	Deletion (het)	1	
	Alternative non-coding exon 1a	Deletion (het)	3	
*CREB3L3*	5’UTR – exon 2	Deletion (het)	1	
	5’UTR – 3’UTR	Duplication	2	
	Exons 3–4	Deletion (het)	1	
	Exon 5	Deletion (het)	1	
*GCK*	5’UTR – alternative exon 1	Deletion (het)	5	[40]
	5’UTR – alternative exon 1	Duplication	1	
*GPIHBP1*	5’UTR – 3’UTR	Deletion (hom)	3	
	Exons 3–4	Deletion (het)	3	
*HNF1B*	5’UTR – 3’UTR	Deletion (het)	3	
*HNF4A*	5’UTR – exon 1	Deletion (het)	1	
	5’UTR – 3’UTR	Deletion (het)	1	[41]
*LDLR*	5’UTR – exon 1	Deletion (het)	1	[32]
	5’UTR – intron 1	Deletion (het)	33	[32]
	5’UTR – exon 2	Deletion (het)	3	[32]
	5’UTR – exon 6	Deletion (het)	1	[32]
	Exons 2–3	Deletion (het)	1	[32]
	Exons 2–6	Duplication	1	[32]
	Exons 2–6	Deletion (het)	10	[32]
	Exons 3–6	Deletion (het)	4	[32]
	Exons 4–6	Deletion (het)	1	
	Exons 5–6	Deletion (het)	1	[32]
	Exon 7	Duplication	1	[32]
	Exons 9–10	Deletion (het)	1	
	Exons 11–12 *	Duplication	1	[32]
	Exons 11–12 *	Duplication	1	[32]
	Exons 11–12	Deletion (het)	1	[32]
	Exons 13–14	Deletion (het)	1	[32]
	Exons 13–15	Deletion (het)	1	[32]
	Exon 16–3’UTR	Deletion (het)	1	[32]
	Exon 17–3’UTR	Deletion (het)	5	[32]
	Exons 18–3’UTR	Deletion (het)	1	[32]
*LDLRAP1*	5’UTR – exon 1	Duplication	1	
*LIPA*	Exon 9–3’UTR	Deletion (het)	1	
	Exon 4	Deletion (het)	2	
*LIPC*	5’UTR – exon 1	Deletion (het)	6	
*LMF1*	Exon 6	Deletion (het)	2	
*LPIN1*	Exons 2–4	Deletion (het)	1	
	Alternative exon 6	Deletion (het)	1	
	Exon 18	Deletion (het)	3	
	Exons 18–19	Deletion (het)	1	
*LPL*	5’UTR – exon 1	Deletion (het)	1	[42]
	5’UTR – exon 2	Deletion (het)	3	[42]
*MFN2*	Exons 4–6	Duplication	1	
*MTTP*	5’UTR – 3’UTR	Deletion (het)	1	
	Exon 10	Deletion (het)	1	
	Exons 10–15	Deletion (hom ×2, het)	3	
*NPC1L1*	Exons 6–10	Deletion (het)	1	
*PCSK9*	5’UTR – 3’UTR	Duplication	5	[43]
*PLIN1*	Exon 3–3’UTR	Duplication	1	
	Exon 8	Deletion (het)	2	
	Exon 2	Deletion (het)	1	
*PNPLA2*	5’UTR – 3’UTR	Duplication	1	
*POLD1*	5’UTR	Duplication	2	
*PPARA*	Alternative non-coding exon 1–3’UTR	Duplication	1	
*WRN*	Exon 3	Deletion (het)	1	
	Exons 9–11	Deletion (het)	1	
	Exon 10	Duplication	1	

“*” indicates that although these CNVs cover the same areas, they were found to have different breakpoints, making each one a unique CNV instance. Abbreviations: *HDL* high-density lipoprotein, *het* heterozygous, *hom* homozygous, *UTR* untranslated region

### Genetic characterization of familial hypercholesterolemia and hypertriglyceridemia

We show the relevant genetic determinants present in the patient subsets for the two most prevalent phenotypes encountered in the clinic (Table 3). Rare SNVs and indels were considered if they occurred in phenotypically relevant genes (i.e. *LDLR*, *PCSK9*, or *APOB* for patients with FH; *LPL*, *APOA5*, *LMF1*, *GPIHBP1*, or *APOC2* for patients with hypertriglyceridemia) had a CADD PHRED-scaled score ≥ 10 plus a predicted deleterious or damaging outcomes by SIFT, PolyPhen2, or MutationTaster, and resulted in a change to the encoded protein’s amino acid sequence. The CNVs described in Table 2 were also used in this characterization.

**Table 3:** Genetic characterization of main phenotypic cohorts sequenced using the LipidSeq panel

	Rare variant	Extreme PS	Overall Genetic Profile			
			Rare variant only	Rare variant and an extreme PS	Extreme PS only	No relevant genetic determinants
Familial Hypercholesterolemia *N* = 924	393 (42.5%)	115 (12.4%)	354 (38.3%)	39 (4.2%)	76 (8.2%)	455 (49.2%)
Hypertriglyceridemia *N* = 1308	312 (23.6%)	428 (32.7%)	227 (17.4%)	82 (6.3%)	346 (26.4%)	653 (49.9%)

The “Rare variant” category includes SNVs, indels, and CNVs; these counts include causative and relevant determinants. An extreme polygenic score was defined as being greater than or equal to the 90th percentile, as calculated using the European subgroup of the 1000 Genomes Project (*N* = 503) [23] The “No related genetic determinants” category refers to patients that had neither a rare variant disrupting a related, canonical metabolism gene, nor an extreme PS. The LDL cholesterol polygenic score calculated in the FH cohort [44] and the triglyceride polygenic score calculated in the hypertriglyceridemia cohort [45] have both been reported previously. Abbreviations: *PS* polygenic score

When considering rare variants—both SNVs and CNVs—and extreme common SNP accumulation, FH patients were more likely to carry a rare variant compared to hypertriglyceridemia patients (46.3% vs. 23.9%), while hypertriglyceridemia patients were more likely to have an extreme accumulation of common SNPs, represented as an extremely high polygenic score compared to FH patients (32.7% vs. 12.4%). Overall, each cohort had ~ 50% of patients with an identifiable, relevant genetic determinant, although the most prominent determinant for FH patients was the presence of a rare variant, while an extreme polygenic score was the most prominent feature in hypertriglyceridemia patients. We are involved in updating ACMG pathogenicity criteria for FH-causing variants and will adjust our diagnostic process when these criteria are published.

## Discussion

We report our clinical and research experience with LipidSeq, a targeted hybrid panel designed for clinical resequencing of genomic loci known to be associated with dyslipidemia and related metabolic traits and disorders. Since 2014, the results from this panel have contributed to 39 publications reporting original scientific findings, including seven on FH [32, 43, 44, 46–49], seven on hypertriglyceridemia [42, 45, 50–54], four on extremes of HDL cholesterol [39, 55–57], and 21 case reports [40, 41, 58–76]. We have published an additional 15 reviews and methods articles related to this work [4, 5, 7, 11–13, 20, 34, 77–83]. Some highlights of outcomes from the use of LipidSeq are summarized in Tables 4 and 5. Several insights emerged, particularly from the 1466 samples acquired from patients of the Lipid Genetics Clinic who were referred for medical care and treatment advice. Sometimes, the research results could be applied directly to inform and guide patient management, especially when confirming a clinical diagnosis of FH and for other less common dyslipidemias (Table 4).

**Table 4:** Selected clinical outcomes using the results from the LipidSeq panel

Suspected disorder	Gene(s) of interest	LipidSeq result	Diagnosis	Number of patients	Clinically relevant outcomes
HeFH	*LDLR, APOB, PCSK9*	Heterozygous rare variant	HeFH	623	- Increased diagnostic certainty
					- Increased likelihood of third-party coverage for PCSK9 inhibitors
HoFH	*LDLR, APOB, PCSK9, LDLRAP1, ABCG5, ABCG8, LIPA*	Bi-allelic rare variants in either *LDLR*, *APOB*, *PCSK9,* or *LDLRAP1*	HoFH	8	- Apheresis needs to be considered as a treatment
					- Higher intensity therapies enter the picture, including lomitapide and mipomersen
					- Investigational treatments include AV8.TBG.hLDLR (RGX-501) gene therapy and anti-ANGPTL3 treatments (evinacumab or IONIS-ANGPTL3-LRx)
		At least one non-null *LDLR* allele	HoFH	3	- A partial response to evolocumab is predicted
		Bi-allelic rare variants in *ABCG5/ABCG8*	Sitosterolemia	3	- Change of clinical diagnosis from HoFH to sitosterolemia
					- Patients switched from standard HoFH treatment to a low plant diet and ezetimibe
		Bi-allelic rare variants in *LIPA*	LALD, CESD or Wolman syndrome	3	- Change of clinical diagnosis from HoFH (or sometimes HeFH), usually in pediatric cases, to LALD [84]
LALD	*LIPA*	Bi-allelic rare variants in *LIPA*	LALD, CESD or Wolman syndrome	3	- Diagnosed patients are eligible for sebelipase (infused lysosomal acid lipase replacement)
ABL/FHBL	*MTTP, APOB, SAR1B, PCSK9, ANGPTL3*	Bi-allelic rare variants in *MTTP*, *APOB* or *SAR1B*	ABL, homozygous FHBL or CRD, respectively	6	- Initiation of lifelong therapy to avert consequences of fat-soluble vitamin deficiencies
					- Fat restricted diet
					- Additional clinical monitoring
Familial chylomicronemia syndrome	*LPL, APOC2, APOA5, GPIHBP1, LMF1*	Bi-allelic rare variants in *LPL, APOC2, APOA5, GPIHBP1,* or *LMF1*	Familial chylomicronemia syndrome	70	- Initiation of lifelong fat restricted diet
					- Potential novel or investigational treatments, such as anti-apo C-III treatments (volanesorsen in Europe or AKCEA-APOCIII-LRx); anti-ANGPTL3 treatments (evinacumab or IONIS-ANGPTL3-LRx)
		Bi-allelic rare variants in *APOC2*	APOC2 deficiency	5	- Potential for investigational apo C-II infusion
Hypoalpha-lipoproteinemia	*LCAT, APOA1, ABCA1*	Bi-allelic rare variants in *LCAT*	LCAT deficiency	2	- Monitoring of renal function
					- Potential for investigational LCAT infusion (ACP-501);
		Bi-allelic rare variants in *APOA1* or *ABCA1*	Apo A-I deficiency or Tangier disease, respectively	4	- Potential for investigational apo A-I infusion (CSL-112)
Lipodystrophy	*LMNA, PPARG*	Heterozygous variants in *LMNA* or *PPARG*	FPLD2 or FPLD3, respectively	130	- Increased monitoring for metabolic syndrome complications
					- Broad-spectrum CVD prevention initiated
					- Possible leptin therapy
MODY	*HNF1A, GCK*	Heterozygous variants usually in *HNF1A* or *GCK*	MODY3 or MODY2, respectively	110	- Switch from insulin to diet and oral hypoglycemic agents particularly in MODY2

Abbreviations: *ABL* abetalipoproteinemia, *CESD* cholesteryl ester storage disease, *CRD* chylomicron retention disease, *CVD* cardiovascular disease, *FHBL* hypobetalipoproteinemia, *FPLD* familial partial lipodystrophy, *HeFH* heterozygous familial hypercholesterolemia, *HoFH* homozygous familial hypercholesterolemia, *LALD* lysosomal acid lipase deficiency, *MODY* maturity-onset diabetes of the young

**Table 5:** Top new insights into dyslipidemia from experience with LipidSeq panel

Insight	Reference
About 50% of referred patients thought to have heterozygous FH with LDL cholesterol > 5 mmol/L (> 190 mg/dL) had a likely causative rare variant. This rises to > 90% for patients with LDL cholesterol > 8 mmol/L (> 310 mg/dL).	[44]
About 10% of rare variants causing HeFH are CNVs of the *LDLR* gene.	[32]
A whole-gene duplication of *PCSK9* is a novel, rare cause of HeFH.	[43]
At least 20% of suspected HeFH patients without rare variants have a high LDL cholesterol polygenic SNP score.	[44]
PCSK9 inhibitors are equally effective in patients with either monogenic or polygenic severe hypercholesterolemia.	[49]
Severe hypertriglyceridemia is mostly defined by rare heterozygous variants and a high triglyceride polygenic SNP score.	[45]
The clinical phenotype in monogenic chylomicronemia is essentially identical irrespective of underlying causative genes and variants.	[50]
Hypoalphalipoproteinemia is usually polygenic, comprising both rare heterozygous variants and a high HDL cholesterol polygenic SNP score.	[55]

Abbreviations: *CNV* copy-number variant, *FH* familial hypercholesterolemia, *HeFH* heterozygous familial hypercholesterolemia, *LDL* low-density lipoprotein, *SNP* single-nucleotide polymorphism

Perhaps the largest impact of DNA-based diagnosis has been upon patients with suspected FH; our laboratory is among the largest contributors of FH variants to the ClinVar database [47]. In contrast to the low yield of FH-causing variants in population-based samples with hypercholesterolemia [85], we find that ~ 50% of referred patients suspected to have FH with LDL cholesterol > 5 mmol/L (> 190 mg/dL) had likely or definite pathogenic variants, which rose to > 90% for patients with LDL cholesterol > 8 mmol/L (> 310 mg/dL) [44]. Furthermore, by simultaneously assessing for CNVs, we increased the diagnostic yield of likely pathogenic *LDLR* variants by ~ 10% [32, 77, 79, 80, 83]. When rare variants were absent, we found at least 20% of patients with suspected heterozygous FH had a high polygenic SNP score [7, 20, 44], indicating accumulated trait-raising alleles at SNP loci associated with LDL cholesterol.

In contrast to FH, most defined cases of severe hypertriglyceridemia (> 30%) were not monogenic, while only 1–2% of cases were diagnosed as familial chylomicronemia syndrome due to biallelic pathogenic variants affecting lipolysis [45]. Among individuals with this monogenic, autosomal recessive condition, there are minimal phenotypic differences when stratifying by causative gene or type of genetic determinant [50]. Among patients with monogenic chylomicronemia, ~ 5% of causative variants were CNVs in the *GPIHBP1* gene [50]. While individuals with monogenic hypertriglyceridemia had higher relative risk of acute pancreatitis than those with multifactorial or polygenic hypertriglyceridemia [51], the absolute number of cases was larger in the latter group, since it is much more prevalent [54]. We showed how the clinical phenotype in some patients with multifactorial hypertriglyceridemia can be as severe as in those with monogenic hypertriglyceridemia [63, 66, 71].

Among patients with severely lowered HDL cholesterol, 2–3% have monogenic disorders (i.e. recessive Tangier disease, LCAT deficiency or apo A-I deficiency) [57]. As with severe hypertriglyceridemia, polygenic factors like heterozygous rare variants with incomplete penetrance and extreme polygenic SNP scores, were much more common among individuals with very low HDL cholesterol [56]. Also, we detected heterozygous large-scale deletions of *ABCA1* in four patients with severely lowered HDL cholesterol, the first report of *ABCA1* CNVs in the context of this phenotype [39].

Beyond characterizing the genetic determinants underlying our patients’ phenotypes, we have also uncovered new mechanisms of disease. In two families with severe FH, we discovered a heterozygous whole-gene duplication of *PCSK9* with extremely high circulating PCSK9 levels [43]. As well, a gain-of-function mutation in *APOC3* was revealed as a new cause for hypertriglyceridemia [68].

Our findings have also been individually impactful for our patients. DNA-based confirmation of the diagnosis of heterozygous FH has helped > 50 patients to obtain private coverage for PCSK9 inhibitor drugs. A pilot pharmacogenetic analysis showed that these agents are equally effective in monogenic and polygenic severe hypercholesterolemia [49]. As well, we diagnosed several patients whose severe hypertriglyceridemia was due to subclinical undiagnosed partial lipodystrophy [59], which altered monitoring and management. Other examples of positive clinical outcomes from use of LipidSeq include: 1) ending the protracted diagnostic odyssey endured by some patients [72, 74, 75]; 2) increasing the diagnostic yield in MODY diabetes by ~ 6% through simultaneous screening for CNVs [40, 76]; 3) switching some patients with *GCK* CNVs (diagnosed with MODY2) from insulin to oral hypoglycemic agents [40]; 4) diagnosing sitosterolemia in patients who were initially diagnosed with homozygous FH, resulting in a dramatic change in management [48]; and 5) ruling out genetic contributions in several patients with severe dyslipidemias due to secondary causes [52, 60, 65].

Sharing research results with patients during follow-up visits has become routine in our practice and has allowed some general observations. We noted early that research findings were frequently illuminating in a clinical context, and as a result we routinely inform patients of their DNA findings on follow-up visits, and cautiously interpret these. The advice many years ago from our ethics review board seems to have anticipated the current importance of disclosure of results to research patients [86]. Since LipidSeq’s content is restricted to lipid disorders, there is no possibility of identifying secondary or incidental findings, except those related to other dyslipidemias.

In the course of reporting findings, we have observed in FH patients that: 1) knowing that there is a causative or relevant genetic finding seems to improve adherence to advice, particularly medication use; 2) when reporting polygenic effects, patients seem to understand the concept better when a simple tally of alleles is presented, compared to a weighted normalized statistic; 3) compliance seems unaffected by whether a monogenic or polygenic basis is communicated (we are undertaking a formal research project to address this issue); and 4) biochemical cascade screening is recommended regardless as to whether the hypercholesterolemia has a monogenic or polygenic basis, since multiple small-effect variants also tend to cluster in closely related family members.

Following from the positive experiences with LipidSeq, we have used it as the template to develop a similar panel for neurodegenerative conditions [82]. Given our experiences, we hope that more individuals, both physicians and researchers, will be able to use similar sequencing strategies for their clinical work and research, respectively. We note that the overall design and procedure used for several commercial dyslipidemia panels offered since about 2016–17 resemble LipidSeq quite closely.

## Conclusions

We have extensively applied our targeted sequencing panel for lipid disorders in a clinical context over several years. LipidSeq has enabled robust detection not just of rare variants underlying monogenic dyslipidemias, but also of CNVs due to high depth of coverage, and polygenic effects because of flexibility to detect common SNPs used in polygenic scores. This additional ability for assessing multiple genetic determinants across multiple genes simultaneously reduces genetic non-diagnoses that might otherwise result from overreliance on methods such as exome sequencing, which are optimized to uncover rare coding SNVs. In contrast, genome sequencing can potentially detect this wide range of variant types, but optimization of bioinformatic and ethical issues are needed first. Our accumulated observations, anecdotes and small case series suggest the value of genetic diagnosis for certain patients and clinical situations. But whether genetic diagnosis alters treatment decisions for the majority of dyslipidemic patients, above and beyond clinical and biochemical criteria alone, requires further study.

## Supplementary information

**Additional file 1: **This additional file contains four supplemental tables, each in its own labelled tab. **Table S1.** Contains a description of each gene on the LipidSeq panel and what disorder or trait it is related to, as well its chromosmal coordinates. **Table S2.** Contains information on our phenotypic categorization of samples that have been sequenced using the LipidSeq panel design. **Table S3.** Contains annotated information on every unique variant we have identified from our entire cohort. **Table S4.** Contains a subset of annotated variants with CADD PHRED scores greater than or equal to 20.

## Data Availability

Patient-level data is unavailable and cannot be shared due to patient privacy and our ethics form. Instead, annotated variant information for each identified rare variant from our total cohort (*N* = 3,262) is available in Additional file 1: Table S3 and Table S4.

## Abbreviations

Apo: Apolipoprotein
CADD: Combined Annotation Dependent Depletion
CNVs: Copy-number variants
ExAC: Exome Aggregation Consortium
FH: Familial hypercholesterolemia
gnomAD: Genome Aggregation Database
GWAS: Genome-wide association studies
HDL: High-density lipoprotein
Indels: Insertions or deletions
LDL: Low-density lipoprotein
MODY: Maturity-onset diabetes of the young
PolyPhen2: Polymorphism Phenotyping version 2
SIFT: Sorting Intolerant from Tolerant
SNPs: Single-nucleotide polymorphisms
SNVs: Single-nucleotide variants
